# The Power of Ideals

**Published:** 1913-03

**Authors:** Jno. S. Turner

**Affiliations:** Dallas, Texas


### The Power of Ideals.

BY MRS. JNO. S. TURNER, DALLAS, TEXAS.

The word "ideal" is defined by Webster as "a mental conception regarded as a standard of perfection."

"The ideal is to be attained," says Fleming, "by selecting and assembling in one whole the beauty and perfections which are usually seen in different individuals, excluding everything defective or unseemly, so as to form a type or model of the species."

None of the laws of nature are so important as those relating to human life, affecting as they do the weal or woe, not only of the individual, but indirectly of society and the welfare of mankind in general.

Since the law of nature decrees that like shall produce like, the character of the child is predetermined by the character of the parents, modified or intensified by their thoughts, feelings and desires during its prenatal existence.

Says Dr. Spurzheim: "He who can convince the world of the importance of the laws of hereditary descent, and induce mankind to conduct themselves accordingly, will contribute more to their improvement than all institutions and all systems of education."

There is much truth in the contention of Oliver Wendell Holmes that to have a healthy child preparations must begin a hundred years before its birth, and yet the vast majority of parents, ignorant of the laws of heredity, give no thought to the training and development of the child prior to its independent existence, many deluding themselves with the belief that the age of accountability is sufficiently early to begin to direct the mind of the child in proper channels.

"It is during the prenatal period of life that education, home influences and the grace of God do their most effectual work in the formation of character," says Prof. N. N. Riddell.

"We are omnibuses in which our forefathers ride," said Dr. Holmes.

This is true, not only of our mental and moral natures, but of our physical natures as well. Because of this physiological fact, it should be the aim and purpose of every prospective parent to so order his or her habits of life as to insure the transmission of the best of them to their offspring. While the mother's influence upon the unborn child is direct, the father's influence, though indirect, is no less potent for good or ill.

The expectant mother of the past was told that in a sudden fright or during excitement if she placed her hand upon any part of her body an imprint of the cause of the mother's emotion would be left upon that identical part of the unborn infant's anatomy. She was taught that ungratified taste or desire for any special food or drink would leave upon the child that longing in an exaggerated degree, to prevent which possible disaster she has indulged herself in improper food and drink, harmful to both mother and child. The acquiring of this knowledge constituted the education of the prospective mother in prenatal influences. In the light of the present agitation of social reform and race improvement, is it too much to venture a prediction that the mothers of the future will be taught that their thoughts, pursuits, habits, acts during the months of gestation, will leave upon their offspring an imprint more deadly than any physical "mark?" That the transmission of life by an intoxicated or semi-intoxicated husband is far more likely to produce a drunkard than any ungratified desire upon the part of the mother. That to indulge in sexual gratification during pregnancy is likely to endow the child with abnormal sexual desires and passions. That weeping, anger, hatred and selfishness will all leave their imprint upon the plastic soul developing within. That to attempt to take the life of even a six-weeks'-old fetus and fail is likely to leave upon that child the imprint of the murderous thought and intent and to produce a criminal.

The writer has on memory's wall the picture of a horror-stricken young wife, a neighbor, who confided to her the various attempts she had made, at the behest of the young husband (brutal wretch!), to destroy the life for which they were responsible. Failing in every attempt, it suddenly dawned upon the prospective mother that these murderous thoughts and efforts might find their way into the brain of her unborn child, and, with horror written upon her countenance, she came to her neighbor for counsel. The remaining months of pregnancy were devoted to efforts to overcome any possible impression upon the fetus during the early stage of its existence. What anguish of soul would this young wife have escaped had she been prepared for motherhood by proper education—which means instruction in potential *parenthood*—for the girl who is *trained* for the profession of motherhood will scarcely take into such a sacred partnership a man capable of such a reprehensible demand.

In happy contrast to this picture is one of a young couple who, shortly after their marriage, moved to a Western ranch, where, the young husband's business requiring his absence from home during most of the daytime, the youthful, expectant mother was left alone, save for the companionship of two faithful young dogs, her sole protection. Threatened miscarriage during the first months of pregnancy gave the prospective parents grave concern, lest they be deprived of the crowning joy of wedlock. About the time the mother became physically conscious of the presence of the little life within, symptoms of threatened miscarriage disappeared and anxiety came to fruition in a buoyant hopefulness. Although no instructions in hereditary tendencies had been received by the young wife, save the warnings referred to above concerning "marks," she knew that children often exhibited to a marked degree characteristics of parents or grandparents. Thus reasoning, she decided that if there be any virtue in maintaining a cheerful disposition in self-control, in kindness to all God's living creatures, in spending her time in useful occupations, that would affect the character of her unborn child, she would put this virtue to the test. Instead of complaining at being left so much alone, she determined by self-control to enjoy solitude and accept the circumstances as an opportunity for mental improvement and physical rest. She refused to permit herself to be frightened by the ominous howling of neighboring wolves, disturbed by the weird cry of the owl or distressed by morbid fears which often accompany loneliness. Besides the time spent in physical exercise incident to the performance of necessary household duties and loving attention bestowed upon the domestic animals about the home, the days were filled with congenial employment varied by the reading of wholesome literature, a small part of each day being devoted to complete rest and meditation. The pleasure enjoyed in the preparation of the little one's wardrobe was shared by the father-to-be, whose praise of the wife's ingenuity in fashioning the little garments and adorning them with her own handiwork added incalculably to her satisfaction.

The reader need not be told that this welcome child, to whose antenatal fostering and culture the mother's energies were cheerfully and lovingly directed, developed into a "cheerful, dutiful, helpful, affectionate and happy child, loving and beloved by all who knew her within and without the family circle."

Dumb animals and little children instinctively recognize her as their friend, as Professor Newton has said of another: "She evidently feels that she has a right to be in the world, that it has a place for her and has a disposition to do all in her power to make the world happier."

"A partnership with God is motherhood;
What strength, what wisdom, what self-control
Belong to her who helps God fashion an immortal soul."

As the mother of Jesus heard a voice saying, "Blessed art thou among women," so may every expectant mother hear a sweet voice in her soul. In the book of Samuel it is recorded that Hannah prayed, and in answer to her soul's agony God gave her Samuel, a child of rare grace and beauty, whose whole life from infancy to old age attested to the faithfulness of his mother and to her dedication of him to God from his inception.

"Before the babe comes, during the quiet months of waiting for its advent, the mother's heart should be as a cloister, hallowed and pure," says Margaret Sangster. "No storm of passion should sweep it, no fretful reluctance should mar its peace."

"The Christian, like the poet," says Henry Drummond, "is born, not made." Nine months of prayer on the part of the mother before the birth of the child is better than nine years of prayer afterwards.

Says Dr. William Lee Howard: "It is in the child's psychic or soul development, its foundation for a good or a weak brain, its moral stability in later life, that the prenatal influence exerts its power. There are, as is well known, an inner self and an outer self. The outer self is our conscious self, the one we *know;* the other self is the one we *feel,* the one which often appears in our waking or sleeping dreams. It is my belief that the inner self has more to do with the soul-growth of the unborn child than the distracted outer self. When the expectant mother sits dreaming of beautiful pictures, when her soul rises in music above her outer self, when she calls up visions of noble deeds, honorable acts, and in ecstasy sees her unborn child fulfilling in reality these dreams, then I believe the influence upon the little growing soul is real, effective, powerful."

On the other hand, a mean, petty, selfish, vain, egotistical mother will impress those traits on her child.

Truly, self-control and the realization of her duty to herself and her unborn child are the prospective mother's first duties. The woman who cannot govern herself, who has no ideal higher than self-gratification, no ambition more worthy than self-aggrandizement or ease, no conception of the cultivation of the immortal soul of her child, is not worthy to become a mother.

If the prospective mother would realize her ideals for her offspring, she must dress healthfully, with no tight or restricting bands about her body; she must have plenty of fresh air, wholesome food, healthful exercise (than which there is nothing better than household duties in moderation), cheerful company and surroundings, access to the best literature (and an appetite for reading and digesting it) and, most of all, have a reverential, prayerful, worshipful state of mind. Furthermore, the highest ideals would be impossible of attainment if the mother be denied at this time the cheerful, loving sympathy and tender solicitude of him who has sworn to "love and cherish, in sickness and in health," for no prospective mother can soar to the heights of ideality without the sympathy and co-operation of him for whom she has forsaken all else. For, as the poet has truthfully expressed it:

"It takes two for a kiss, but one for a sigh;
Twain by twain we marry, one by one we die;
Joy is a partnership, grief weeps alone;
Many guests had Cana, Gethsemane but one."

---

Below will be found a number of questions that have been asked our hookworm specialist—Dr. Boerner—not once but many times and not by one person but by many. Dr. Boerner has proven most efficient, and deserves the commendation and assistance of all thinking people.

Question—What are hookworms?

Answer—They are small, round, white worms about one-half to three-quarters of an inch in length, and about the size of No. 8 spool thread.

Q.—Where do they live?

A.—They live only in the small intestine (bowel). The grown worms cannot live outside the human body.

Q.—What do the worms do in the body?

A.—(a) They suck blood from the mucous (lining) membrance of the bowel. (b) They inject a poison into the circulation. (c) The female worms lay eggs, which pass out in the stools.

Q.—What becomes of the eggs which pass out in the stools?

A.—In from a few hours to a few days they hatch out young worms which grow two or three days and shed their skin; they eat and grow for three or four days more, when they cast their skin again and are called "encysted embryos."

Q.—Can these embryos be seen with the naked eye?

A.—No. They can be seen only with the aid of the microscope.

Q.—How is the hookworm disease contracted?

A.—By going barefooted or wearing leaky shoes on damp ground that has been polluted by stools which contain hookworm eggs and embryos.

Q.—How do the embryos get into the body and cause the disease?

A.—If a barefoot child steps on polluted soil, the little embryos crawl through the skin and get into the blood stream and are carried through the heart to the lungs. They are then coughed up and swallowed and finally make their way through the stomach to the small bowel, where they find a place suitable for their development.

Q.—How long is it from the time they first crawl through the skin until they are ready to suck blood and inject their poison?

A.—About seven weeks.

Q.—Do the hookworms multiply in the bowels?

A.—No, they do not. Each worm in the bowel represents an embryo which crawled through the skin.

Q.—How many worms have been found in one person?

A.—In one person over 3000 worms were found.

Q.—What are some of the symptoms of hookworm disease?

A.—Pale skin, puffiness about the eyes and face, indigestion, pain in the region of the stomach, headache, dizziness, fluttering of the heart, a tired and worn-out feeling, shortness of breath, diarrhea or constipation, sores on the legs, dirt-eating, stunted growth, and swelling of the feet and legs, or general dropsy. There are many cases which are so mild that but few, if any, symptoms are found.

Q.—How can hookworm disease be prevented and stamped out?

A.—(a) By wearing good shoes. (b) By curing all persons who have the disease. (c) By using a sanitary privy.

These questions will be continued in the April issue. The next feature of the question will be "soil pollution." Be sure and watch for it; it's worth while that you should know about these things.

---

Danger in the Soft Drinks.—Pure Food Commissioner Abbott has ruled and given orders that those who dispense what they call soft drinks shall not only wash every glass after use, but dry it on a clean napkin, the same as is done by those who dispense milk, beer, high-balls or any other drink. A very sensible order.

